# Effect of Shift Work on Cognitive Function in Chinese Coal Mine Workers: A Resting-State fNIRS Study

**DOI:** 10.3390/ijerph19074217

**Published:** 2022-04-01

**Authors:** Fangyuan Tian, Hongxia Li, Shuicheng Tian, Jiang Shao, Chenning Tian

**Affiliations:** 1 Institute of Safety Management & Risk Control, School of Safety Science and Engineering, Xi’an University of Science and Technology, Xi’an 710054, China; 18120089008@stu.xust.edu.cn (F.T.); 19120089021@stu.xust.edu.cn (C.T.); 2 Institute of Safety & Emergency Management, School of Safety Science and Engineering, Xi’an University of Science and Technology, Xi’an 710054, China; 3 School of Management, Xi’an University of Science and Technology, Xi’an 710054, China; 4 School of Architecture & Design, China University of Mining and Technology, Xuzhou 221116, China; shaojiang@cumt.edu.cn

**Keywords:** Chinese coal mine workers, fNIRS, functional connectivity, cognitive ability, shift work

## Abstract

Aim: Pilot study to examine the impact of shift work on cognitive function in Chinese coal mine workers. Background: Shift work is commonly used in modern industries such as the coal industry, and there is growing concern over the impact that shift work has on miners’ work performance and personal well-being. Method: A total of 54 miners working three shifts (17 in morning shift, 18 in afternoon, and 19 in night shift) participated in this exploratory study. A resting-state fNIRS functional connectivity method was conducted to assess the cognitive ability before and after the work shift. Results: Results showed significant differences in cognitive ability between before and after the work shifts among the three-shift workers. The brain functional connectivity was reduced ranking as the night, afternoon, and morning shifts. Decreased brain functional connectivity at the end of the working shift was found compared with before in the morning and afternoon shifts. Opposite results were obtained during the night shift. The resting-state functional brain networks in the prefrontal cortex of all groups exhibited small-world properties. Significant differences in betweenness centrality and nodal local efficiency were found in the prefrontal cortex in the morning and night shifts. Conclusions: The current findings provide new insights regarding the effect of shift work on the cognitive ability of Chinese coal mine workers from the view of brain science.

## 1. Introduction

In modern industries, represented by the coal industry, miners are required to work alternating morning, afternoon, and night shifts (three-shift) within 24 h to ensure the normal operation of the company’s production. According to the “China Coal Industry Development Annual Report 2020”, there are over 2 million shift coal mine workers in China [1]. However, shift work not only affects the physical and mental health of shift workers but also reduces their work efficiency and personal well-being [2,3]. Lack of adequate or regular sleep affects alertness, reaction times, eye-hand coordination and concentration, and other cognitive abilities of shift miners, resulting in fatigue, degraded work performance, unsafe behaviors, and causing accidents [4,5,6,7]. Due to the unique dangerous and underground working environment, coal mine workers have been considered one of the riskiest jobs [8]. Evidence shows that approximately one-half of major accidents occurred in coal mines in China, and 95% of them were caused by human factors, especially unsafe behavior [9,10]. To further reduce the occurrence of unsafe behavior, enhance the safety management of coal mine companies and protect the physical and mental health of coal mine workers, it is necessary to investigate the cognitive performance of shift coal mine workers before and after their shifts [11].

Cognitive functions, such as executive functions, working memory, attention, and information processing speed, are the important guarantee of safe work [12,13]. Laboratory studies provide evidence for cognitive functions declines associated with shift work in nurses, doctors, miners, and petrochemical control room operators [12,13,14,15,16,17,18]. Long-time shift work or irregular circadian rhythm could decrease mental and behavioral performance such as declined working memory, inattentiveness, fatigue, sleepiness, lower sleep quality, longer response time, reduced alertness level, decreased ability to learn and recall new facts, irritability, bad mood, reduced communication skills [3,12,13,15,19]. Moreover, it could lead to increased risk and unsafe behavior, operator error rates, and cause accidents [11,13,15,19]. Neuropsychological performance, such as memory performance, tends to decrease with the increase in the duration of exposure to shift work [12].

In recent years, an increasing number of scholars have focused on the effects of shift work on the cognitive functions of coal mine workers. Glenn Legault et al. summarized the effect of sleep deprivation, shift working, and heat exposure on miners and combined objective measures (actigraphy) and subjective measures (Karolinska and Epworth sleepiness scales) to measure the cognitive consequences of shiftwork for miners [14,20]. Sally A. Ferguson et al. investigated the impact of work- and sleep-related factors on an objective measure (Actiwatch) of response time in a field setting [21]. Rebecca Jane Loudoun et al. found that shift work, aging, and lack of control at work increased sleep problems in miners [22]. Haimiao Yu et al. deployed a reaction time test and “psychometric fatigue assessment scale” to measure the differences in mental fatigue between day-shift and night shift coal mine workers [23]. Camila Pizarro-Montaner et al. analyzed the sleep quality and physical activity of coal mine shift workers in high altitudes [24]. Based on Pittsburgh Sleep Quality Index (PSQI), Xiao-Chuan Zhao et al. investigated the effects of shift work on sleep and cognitive function in miners [25]. Lavigne et al. applied an actigraph and vigilance scale to assess the change in sleep and vigilance of underground miners during long periods of extended shifts [26].

However, most scholars have explored the effect of shift work on cognitive function based on subjective and/or behavioral measures such as behavioral experiments, neuropsychological tests, psychological scales, questionnaires, and wearable devices [8,12,14,27,28]. Subjective measures are highly dependent on the subjects’ self-will and awareness of their cognitive status [29]. Behavioral measures are limited in the range of information they provide and may interfere with task performance, inducing additional load [30]. Compared with subjective and behavioral measures, as a reliable quantitative assessment, neurophysiological measurements such as resting-state functional near-infrared spectroscopy (fNIRS) could be introduced to further explore the effect of shift work on cognitive function. In recent years, fNIRS has been considered an emerging imaging technique in a safe, cost-effective, comfortable, and portable way to investigate safety problems [31,32,33]. In neuroscience, the prefrontal cortex (PFC) is an accepted key brain area of human cognitive functions [34]. The fNIRS resting-state functional connectivity (RSFC) has been proved to be a novel and advanced method to assess and monitor cognitive states such as fatigue, mental workload, vigilance, sustained attention, and error recognition [35].

Thus, to fully comprehend the neuropsychological mechanism of the effect of shift work on cognitive function in Chinese coal mine workers, the current study examined a resting-state functional near-infrared spectroscopy (rs-fNIRS) study in the PFC of 54 Chinese coal mine workers and evaluated the functional connectivity before and after their shifts in three-shift work in a real environment to further explore the cognitive function changes in three-shift coal mine workers from a brain science standpoint.

## 2. Materials and Methods

### 2.1. Demographic Information of the Subjects

A total of 60 Chinese coal mine workers from Shaanxi Coal Group Northern Shaanxi Mining Hongliulin Company, one of the largest modern coal mines in China, were randomly selected to participate in this experiment. After excluding the bad data such as heavy head movement, the fNIRS data of 54 miners before and after the shift were obtained in this experiment. Among them, 17 miners were morning shift workers (8:00–16:00), 18 miners were afternoon shift workers (16:00–24:00), and 19 miners were night shift workers (24:00–8:00). The morning and afternoon shifts are coal mining production shifts, while the night shift is maintenance and repair shifts. Although the working hours of the shift miners are 8 h a day, the miners have to arrive 2 h before the shift to prepare for the pre-shift meeting, change their equipment and take the shuttle bus down the mine, and after the shift, they also need to have a post-shift meeting, take a shower and change their clothes. Therefore, each miner works more than 10 h a day. In order not to affect the normal work of the miners, we choose to conduct the pre-shift experiment before the pre-shift meeting and the post-shift experiment after the miners go up to the mine to wash up. For convenience, the morning shift workers were identified as group 1, the afternoon shift workers as group 2, and the night shift workers as group 3. Specifically, before the morning shift work was defined as B1, after the morning shift work was defined as A1. Similarly, before the afternoon shift work was defined as B2, after the afternoon shift work was defined as A2; before the night shift work was defined as B3, and after the night shift work was defined as A3.

The demographic information of these coal mine workers is detailed in Table 1 and Table 2. The average age of the participants was 36.06 ± 7.42 years, the average height was 172.63 ± 4.71 cm, and the average weight was 69.93 ± 8.36 kg. Participants were all right-handed, and none of them had a history of neurological illness or psychiatric disorders. Further, it was forbidden to drink alcohol or caffeine, and other sensitive products 24 h before the experiment. During the 5 min rs-fNIRS experiment, the participants were asked to remain still and stare at the center cross of the screen without falling asleep. The light and temperature (25 °C) of the experimental room were kept constant throughout the whole experiment.

Before the experiment, all participants were fully informed of the contents of the rs-fNIRS experimental program. All procedures followed the Human Ethics Committee of Xi’an University of Science and Technology and met the ethical standards of the 1975 Helsinki Declaration.

### 2.2. Data Acquisition

**Figure 1:**
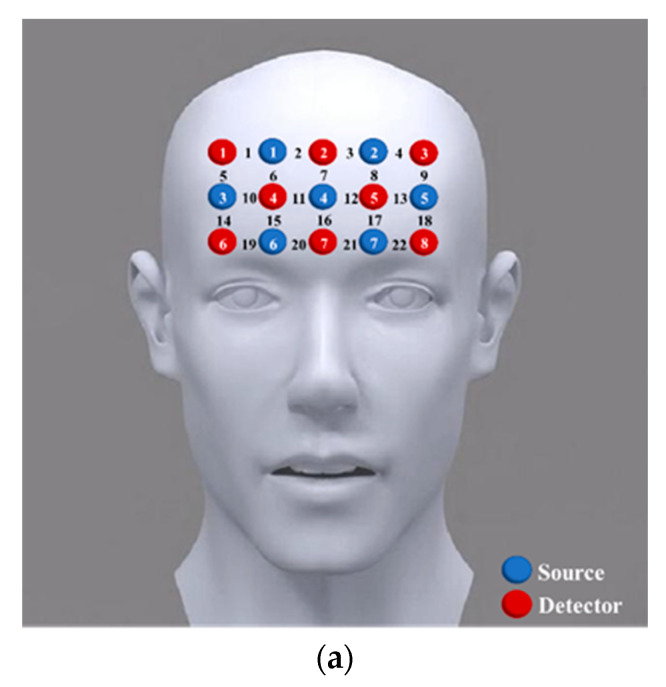

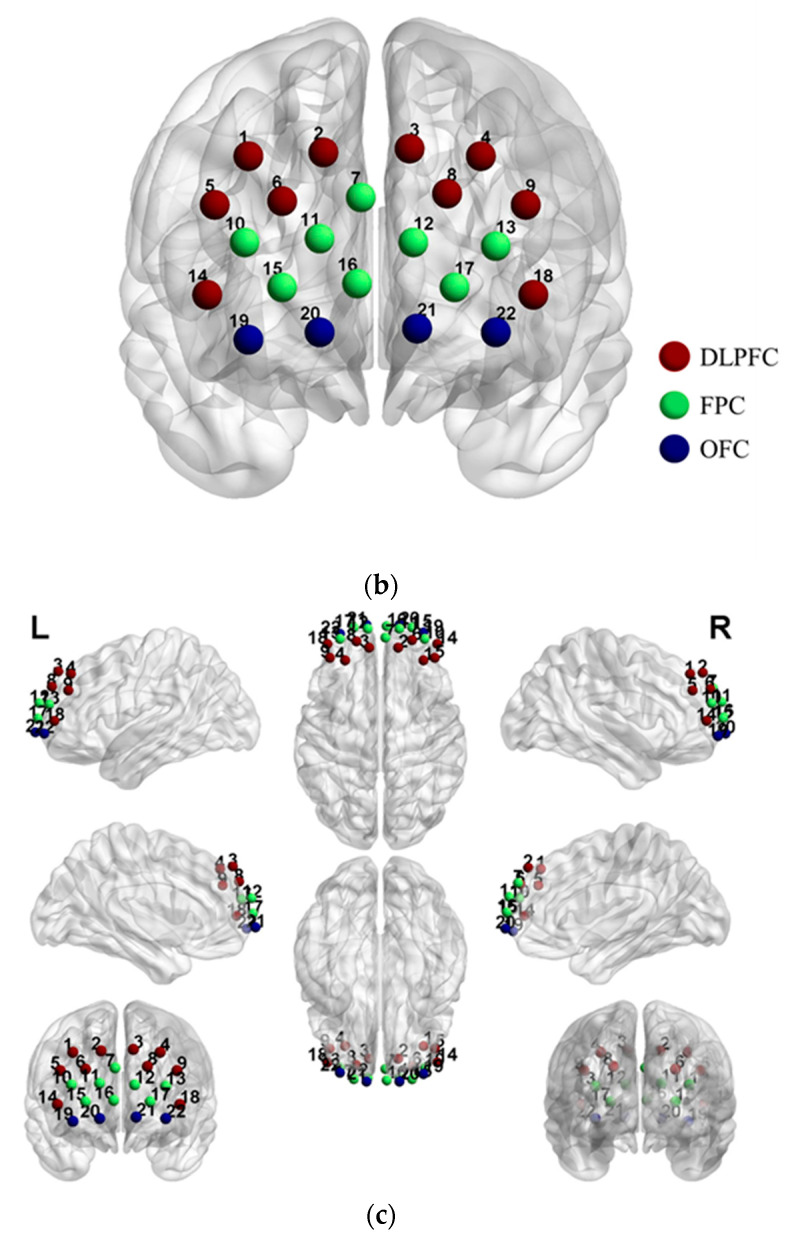
(**a**) Positions of fifteen optodes (eight sources and seven detectors) and fNIRS channels. (**b**) Fifteen optodes were attached to the prefrontal cortex forming 22 channels in frontal view. (**c**) The 3D MNI coordinates of 22 channels in a different view. DLPFC means dorsolateral prefrontal cortex, FPC means frontopolar cortex, OFC means orbitofrontal cortex.

In this study, the hemodynamic responses were measured using a near-infrared optical imaging system (LABNIRS; Shimadzu Corporation, Kyoto, Japan) with a sampling rate of 7.4074 Hz. As shown in Figure 1, the system was equipped with 7 sources and 8 detectors emitting light at two different wavelengths (690 and 830 nm), defining 22 channels, covering the PFC. The distance between a pair of a source and a detector was 30 mm. To ensure accuracy of positioning, detector 7 was placed perpendicular to the tip of the nose and flush with the eyebrows.

The locations of all fNIRS channels were measured using a 3D digitizer system (FASTRAK; Polhemus, Colchester, VT, USA) after the experiment. The system had its origin at the center of the chin with nasion (Nz), right preauricular points (AR), left preauricular points (AL), and central zero (Cz) as reference points [36]. The locations of the fifteen fNIRS optodes were obtained according to the origin and the four reference points. The coordinate of an fNIRS channel was computed based on the locations of the sources and detectors using the MATLAB toolbox NIRS-SPM (The MathWorks Inc., Natick, MA, USA) [37]. As shown in Table 3, the estimated mean locations of 22 channels were obtained based on Brodmann Areas’ anatomical information. Consistent with previous studies, PFC was chosen to be the main regions of interest (ROIs) of this study, including the dorsolateral prefrontal cortex (dlPFC) (CH01, CH02, CH03, CH04, CH05, CH06, CH08, CH09, CH14, and CH18), the frontopolar cortex (FPC) (CH07, CH10, CH11, CH12, CH13, CH15, CH16, and CH17) and the orbitofrontal cortex (OFC) (CH19, CH20, CH21, and CH22) [34,38].

### 2.3. Data Preprocessing

In this study, the fNIRS signals recorded during the whole experiment were analyzed using MATLAB by our script (R2013b, MathWorks, Natick, MA, USA). Firstly, a bandpass filter (0.02–0.1 Hz) was applied to detrend and reduce high-frequency noise due to respiration, cardiac pulsations, and optodes’ movements [39,40,41]. Secondly, wavelet-based correction of extreme values was performed by Wavelab850 toolbox to reduce head movements and surface noise and set the parameters as: Mother wavelet: ‘Vaidyanathan’, support: 10, threshold:  0.0001, alpha: 0.1 [42]. Thirdly, the mean value of the differential pathlength factor ($DPF_{mean}=6.53\;\pm \;0.99$) was adopted [43]. Finally, the hemodynamic responses were computed from the processed light intensity using the modified Beer–Lamberts’ law (MBLL) [44]. Consistent with previous studies, oxy-Hb signals were chosen as the research objects of this study because of their better sensitivity and robustness to the changes associated with regional cerebral blood flow [45,46].

### 2.4. Resting-State Functional Connectivity Analysis

#### 2.4.1. Pearson’s Correlation Coefficient and *T*-Test

Pearson correlation coefficients (*COR*) correlation matrices were conducted to measure the strength of the functional connection between brain channels for each coal mine worker on each shift [47]. The *COR* matrix for each shift is the average of the *COR* matrix between the 22 × 22 channels of each coal mine worker in the 5 min resting state. In this study, the mean time course for one participant as $X=\left(x_{i}{\left(t\right)}_{t=1,2,\ldots N}\right)$, where $x_{i}{\left(t\right)}_{t=1,2,\ldots N}\;$ is the mean time series of the ith region, we defined *COR* as [48]:

(1) $$COR\left(x_{i},x_{j}\right)=\frac{\sum_{t=1}^{N}\left[x_{i}\left(t\right)-\bar{x_{i}}\right]\left[x_{j}\left(t\right)-\bar{x_{j}}\right]}{\sqrt{\sum_{t=1}^{N}{\left[x_{i}\left(t\right)-\bar{x_{i}}\right]}^{2}}\sqrt{\sum_{t=1}^{N}{\left[x_{j}\left(t\right)-\bar{x_{j}}\right]}^{2}}}$$

For further interpretation of the difference among these three groups of functional connectivity, the *COR* matrix was binary transformed. Similar to previous studies, the threshold was set as 0.7 [33]. In this study, it is defined that if COR>0.7, COR=1; otherwise, COR=0 [33]. The paired *t*-test was used to compare before and after the shift work in three groups. Since the statistical test was conducted for 22 networks independently, false discovery rate (*FDR*) correction was adopted to eliminate the multiple comparison problems (*q* < 0.05) [49]. All statistical calculations were performed by SPSS 26.0 (SPSS Inc., Chicago, IL, USA) (*p* < 0.05).

#### 2.4.2. Brain Network Analysis

To further quantify the functional connectivity for complex network analysis, a graph theory approach was adopted to compare the rs-fNIRS topological properties of the brain networks between 22 × 22 channels before and after work shifts in three coal mine workers groups [50,51]. The graph theory analysis has multiple important parameters:

Clustering coefficient ($C_{net}$): the local efficiency in information transfer of the network and generally defined as [52]:

(2) $$C_{net}=\frac{1}{n}\underset{i\in N}{\sum }C_{i}=\frac{1}{n}\underset{i\in N}{\sum }\frac{2t_{i}}{k_{i}\left(k_{i}-1\right)}$$

where $k_{i}$ is the number of links connected to a node, $t_{i}$ is the number of triangles around a node *i*.

Global efficiency ($E_{global}$): the ability of information transmission of the global network and generally defined as [52]:

(3) $$E_{global}=\frac{1}{N\left(N-1\right)}\underset{i,j,i\neq j}{\sum }\frac{1}{d_{ij}}$$

where $d_{ij}$ is the shortest path length between nodes *i* and *j.*

Local efficiency ($E_{loc}$): the ability of information transmission of the local network and generally defined as [52]:

(4) $$E_{loc}=\frac{1}{n}\underset{i\in N}{\sum }E_{loc,i}=\frac{1}{n}\underset{i\in N}{\sum }\frac{\sum_{j,h=N,j\neq i}a_{ij}a_{ih}{\left[d_{jh}\left(N_{i}\right)\right]}^{-1}}{k_{i}\left(k_{i}-1\right)}$$

Shortest path length ($L_{p}$): the overall routing efficiency of a graph and generally defined as:

(5) $$L_{p}=d_{ij}=\underset{a_{uv}\in gi↔j}{\sum }a_{uv}$$

where $g_{i↔j}$ is the shortest path between *i* and *j*.

Betweenness centrality ($B_{c}$): the influence of an index node over information flow between all other nodes in a network and generally defined as [48]:

(6) $$B_{ch}=\frac{1}{\left(n-1\right)\left(n-2\right)}\underset{\begin{array}{c} i,j\in N\\ h\neq j;h\neq i;j\neq i \end{array}}{\sum }\frac{d_{ij}\left(i\right)}{d_{ij}}$$

where $d_{ij}$ is the shortest path length between nodes *i* and *j.*

Small-world parameters (σ): modular processing and efficient transmission of information between network characteristics modules and generally defined as [53,54]:

(7) $$\sigma =\frac{\gamma }{\lambda }$$

where $\gamma =C_{net}/C_{ran},$ $\lambda =L_{net}/L_{ran}$, C, and $C_{ran}\;$ are the clustering coefficients, *AL* and $AL_{ran}$ are the characteristic path lengths, $C_{ran}$ and $L_{ran}$ are the average clustering coefficient and characteristic path length of the real network and a random network (σ≫1) [52].

These parameters were calculated by the GRETNA toolbox on MATLAB [55]. According to previous studies, a range of continuous threshold values *T* (T∈(0.1:0.1:0.9)) were adopted to construct the brain networks [45,56]. A total of 100 matched random networks were generated to compute the ratios of the above parameters between the real brain functional networks [50,57]. The paired *t*-test was used to confirm the differences between before and after the shift work in three groups (*p* < 0.05).

## 3. Results

### 3.1. Demographic Information

Table 1 illustrates the demographic information for the total of 54 coal mine workers and three subgroups. Overall, the chi-square test showed that there were no significant differences in demographic information between the three groups of coal mine workers (*p* < 0.05). In particular, the mean length of service of all participants was approximately 10 years (9.91 ± 7.81), while the mean height of them was 172.63 ± 4.71. The mean age of these participants was 36 years (36.06 ± 7.42) old, while the mean weight of them was nearly 70 kg (69.93 ± 8.36).

The results of the one-way ANOVA showed that there was a significant difference between coal mine workers’ brain functional connectivity and marital status (*p* = 0.038) and education information (*p* = 0.019). Unfortunately, there was no significant difference between coal mine workers’ brain functional connectivity and the mean length of service, height, age, and weight among subgroups.

Table 2 showed that the marital status and education information of 54 coal mine workers and three subgroups. Almost 90% of these coal mine workers were married, and more than half of them were graduated from high school.

### 3.2. Pearson’s Correlation Coefficient and T-Test

**Figure 2:**
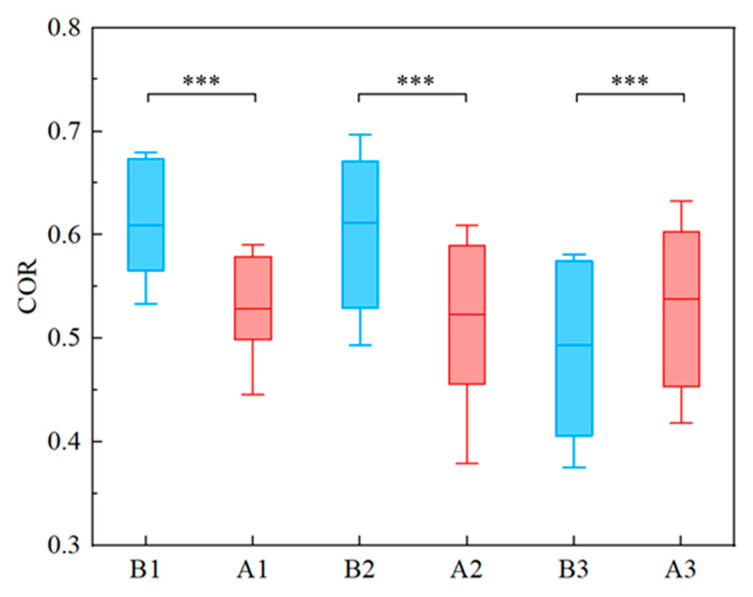
Comparison of the mean *COR* before and after the work shifts among three groups. Note: *** indicates that the results passed paired *t*-test (*p* < 0.001).

Figure 2 demonstrates the mean *COR* before and after shifts for three groups of coal mine workers. In which red represents before shifts and blue denotes after shifts, and the horizontal line is the mean value. As shown in Figure 2, the mean *COR* values of all three groups of coal mine workers before and after their shifts were significantly different (*p* < 0.001). Of these, the mean *COR* of group 1 was declined from before shifts ($\bar{COR_{B1}}=0.6096$) to after shifts ($\bar{COR_{A1}}=0.52895$). A similar reduction in the mean *COR* was obtained in group 2 between before shifts ($\bar{COR_{B2}}=0.6112$) and after shifts ($\bar{COR_{A2}}=0.5234$). Unlike group 1 and group 2, the mean *COR* was increased from before shifts ($\bar{COR_{B3}}=0.4932$) to after shifts ($\bar{COR_{A3}}=0.5384$). Combining the six states of the three groups of coal mine workers before and after their shifts, the mean *COR* was ranked from highest to lowest: $\bar{COR_{B2}}$, $\bar{COR_{B1}}$, $\bar{COR_{A3}}$, $\bar{COR_{A1}}$, $\bar{COR_{A2}}$, $\bar{COR_{B3}}$.

**Figure 3:**
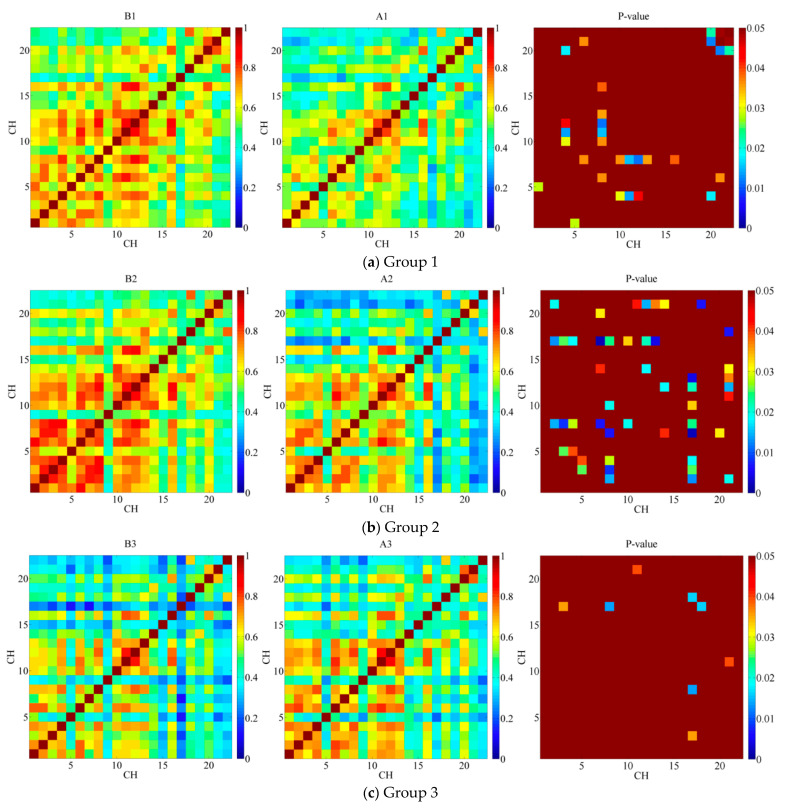
Functional connection matrix and *p*-value between each two channels of 3 shifts coal mine workers (*p* < 0.05). (**a**) Group 1. (**b**) Group 2. (**c**) Group 3.

Figure 3 shows the 22 × 22 correlation matrices and the paired *t*-test results for three shifts of coal mine workers (*p* < 0.05). Each grid denotes the correlation coefficient of a pair of channels (COR∈[0,1]). *COR* indicates the degree of correlation between pairs of channels. If COR→1, there is significantly related to the activation between pairs of channels. As shown in the legend, a redder grid indicates a more correlated pair of channels, while a bluer one is less correlated. Overall, all three groups of coal mine workers had significantly different *COR* matrices before and after their shifts.

It is shown in Figure 3a that the functional connectivity of B1 was larger than that of A1 (*p* < 0.05). That is, compared to before the work shift, prefrontal functional connectivity was significantly decreased after the work shift in group1. These differences are concentrated in the following four categories: (a) the functional connectivity within dorsolateral prefrontal cortex (dlPFC): CH01-05 (*p* = 0.0285), CH06-08 (*p* = 0.0359); (b) the functional connectivity between dlPFC and frontopolar cortex (FPC): CH04-10 (*p* = 0.0304), CH04-11 (*p* = 0.0134), CH04-12 (*p* = 0.0446), CH08-10 (*p* = 0.0367), CH08-11 (*p* = 0.0155), CH08-12 (*p* = 0.0113), CH08-13 (*p* = 0.0357), CH08-16 (*p* = 0.0389); (c) the functional connectivity between dlPFC and orbitofrontal cortex (OFC): CH04-20 (*p* = 0.0177), CH06-21 (*p* = 0.036); (d) the functional connectivity within OFC: CH20-21 (*p* = 0.0124), CH20-22 (*p* = 0.0226), CH22-21 (*p* = 0.0488).

Similar to group 1, Figure 3b shows that the functional connectivity of B2 was also larger than that of A2 (*p* < 0.05). Compare to group 1, the paired *t*-test results showed that the difference in functional connectivity before and after the work shift is greater in group 2. Specifically, these differences are clustered in the following five categories: (a) the functional connectivity within dlPFC: CH02-08 (*p* = 0.0148), CH04-05 (*p* = 0.0394), CH04-09 (*p* = 0.0286); (b) the functional connectivity between dlPFC and FPC: CH02-17 (*p* = 0.0126), CH03-17 (*p* = 0.0251),CH04-17 (*p* = 0.0181), CH07-14 (*p* = 0.0409), CH08-17 (*p* = 0.0235), CH12-14 (*p* = 0.0187); (c) the functional connectivity within FPC: CH07-17 (*p* = 0.0023), CH10-17 (*p* = 0.0333), CH12-17 (*p* = 0.0219), CH13-17 (*p* = 0.0040), CH13-21 (*p* = 0.0370); (d) the functional connectivity between FPC and OFC: CH07-20 (*p* = 0.0313), CH11-21 (*p* = 0.0419), CH12-21 (*p* = 0.0141); (e) the functional connectivity between dlPFC and OFC: CH02-21 (*p* = 0.0188), CH14-21 (*p* = 0.0311), CH18-21 (*p* = 0.0069).

In contrast to the other two groups, Figure 3c shows that the functional connectivity of B3 is less than A3 with the fewest difference pairs (*p* < 0.05). There are only three categories of these differences: (a) the functional connectivity between dlPFC and FPC: CH02-07 (*p* = 0.0388), CH03-17 (*p* = 0.0449), CH06-07 (*p* = 0.0249), CH07-09 (*p* = 0.0456); (b) the functional connectivity within FPC: CH07-11 (*p* = 0.0132), CH07-12 (*p* = 0.0076); (c) the functional connectivity within dlPFC: CH01-04 (*p* = 0.0441).

**Figure 4:**
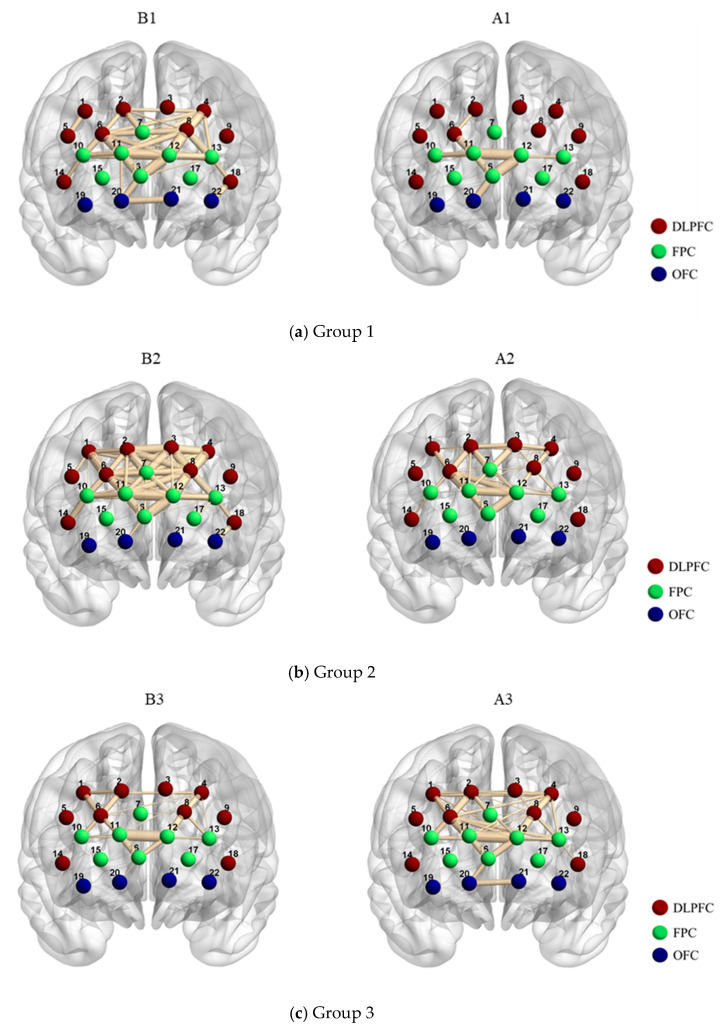
The group connectivity was calculated from oxy_Hb before and after work in 3 shifts of coal mine workers. Only connections with absolute z-values greater than 0.7 were displayed. Brain map images were generated using BrainNet Viewer software [58]. (**a**) Group 1. (**b**) Group 2. (**c**) Group 3.

Figure 4 shows the PFC functional connectivity brain maps of three groups of coal mine workers in different states with COR∈[0.7,1]. As shown in the illustration, the 22 circles represent CH01 to CH22, dlPFC is in brown, FPC is in green, and OFC is in blue. The thickness of the lines between channels represents the value of the *COR*, with thicker lines indicating higher functional connectivity between pairs of channels. In general, group 1 and group 2 generally had reduced functional connectivity after the shift than before, while the opposite was true for group 3. As shown in Figure 4a, the functional connectivity existed among dlPFC, FPC, and OFC before the work shift, while it was decreased and mainly concentrated in the FPC after the work shift in group 1. The functional connectivity of both before and after work shifts for group 2 were mainly focused on dlPFC between FPC and decreased after work shifts (Figure 4b). For group 3, the functional connectivity was mainly concentrated in dlPFC and FPC before the work shift, whereas it was enhanced and existed among dlPFC, FPC, and OFC after the work shift (Figure 4c).

### 3.3. Brain Network Analysis

**Figure 5:**
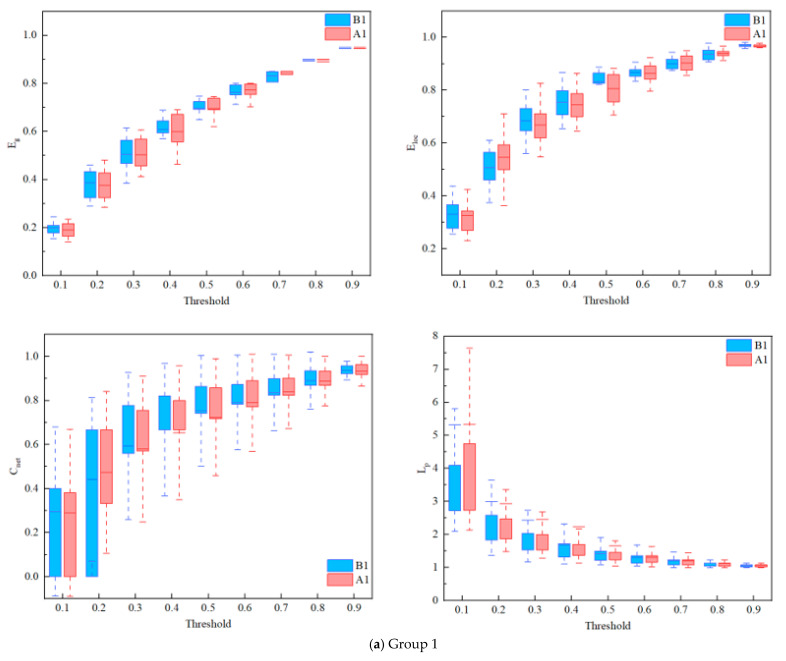

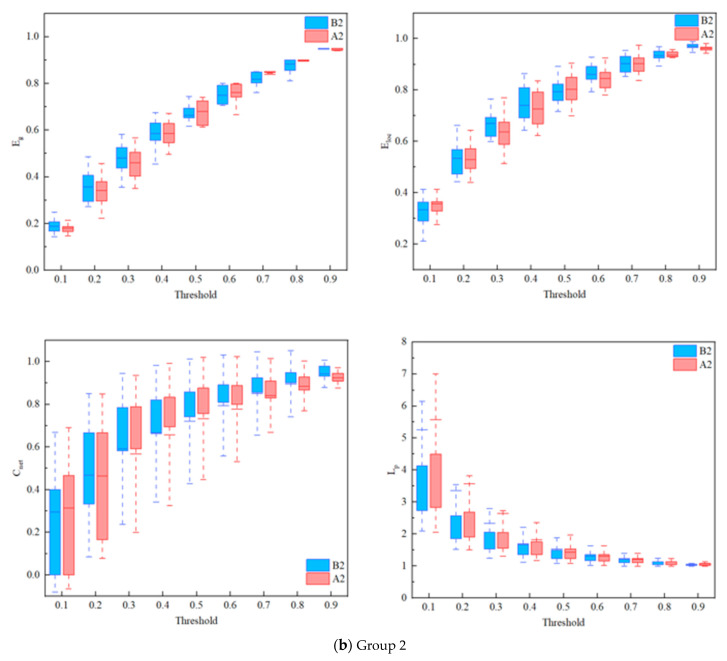

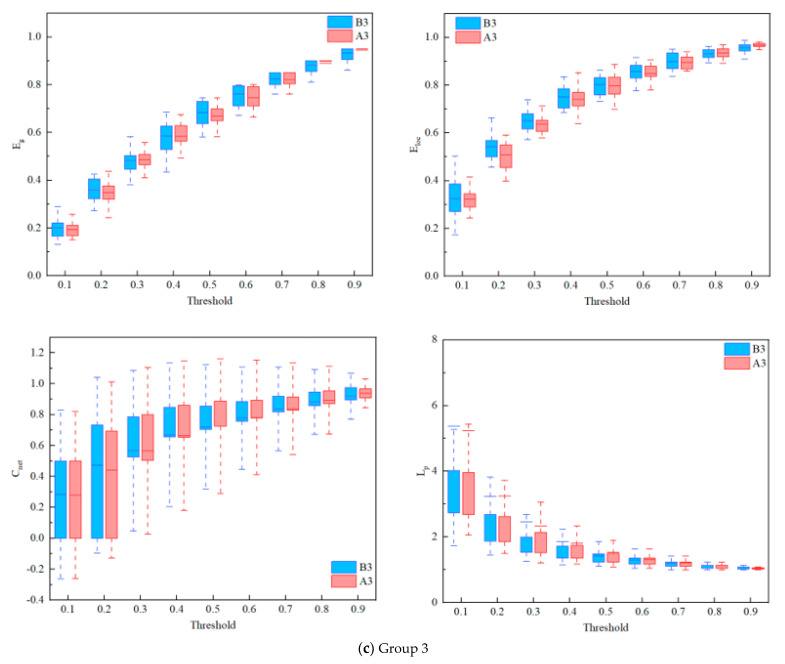
Comparison of the brain network among 3 shifts coal mine workers between before and after shift work: network efficiency. The horizontal axes show the threshold values (T∈(0.1:0.1:0.9)), and the vertical axes show the network properties indexes (*p* < 0.05). (**a**) Group 1. (**b**) Group 2. (**c**) Group 3.

For the brain network analysis, the threshold of this study was conducted to a range of (0.1:0.1:0.9). As shown in Figure 5, $C_{net}$, $E_{global}$, $E_{loc},$ and $L_{p}$ were chosen to denote the network efficiency in these three groups before and after the work shift. Consistent with previous studies, for all three groups of coal mine workers before and after their shifts, $C_{net}$, $E_{global}\;$, and $E_{loc}$ increased with the threshold, while $L_{p}$ decreased as the threshold increased [50,51]. The results of these network characteristics above showed that there were stable small-world characteristics of PFC functional networks in their before and after work shifts for all three groups of coal mine workers [51,59].

**Figure 6:**
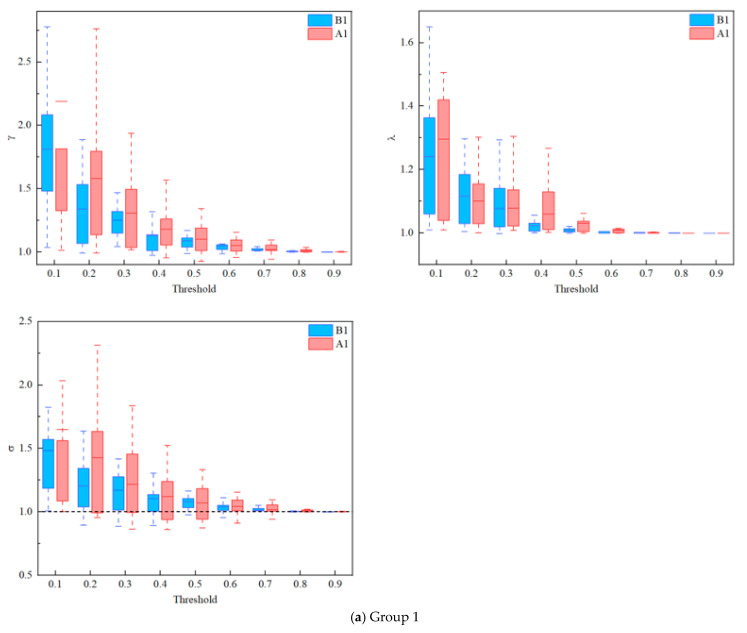

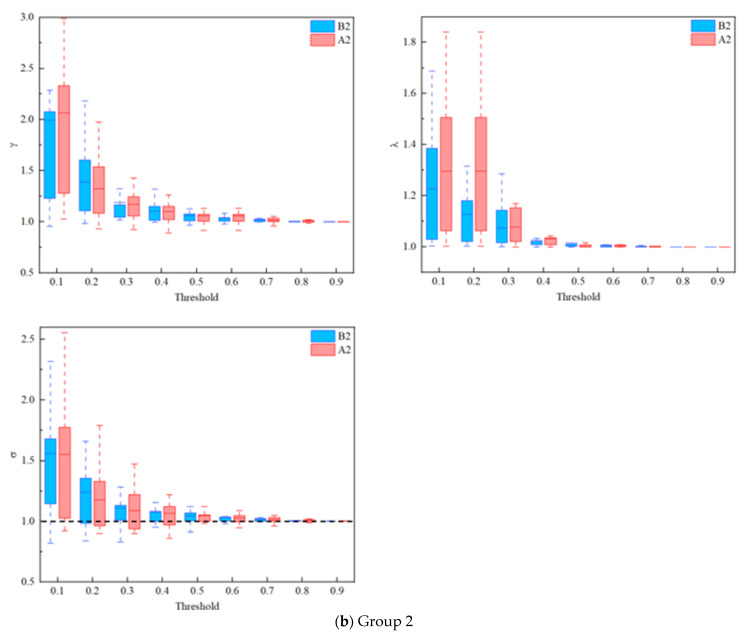

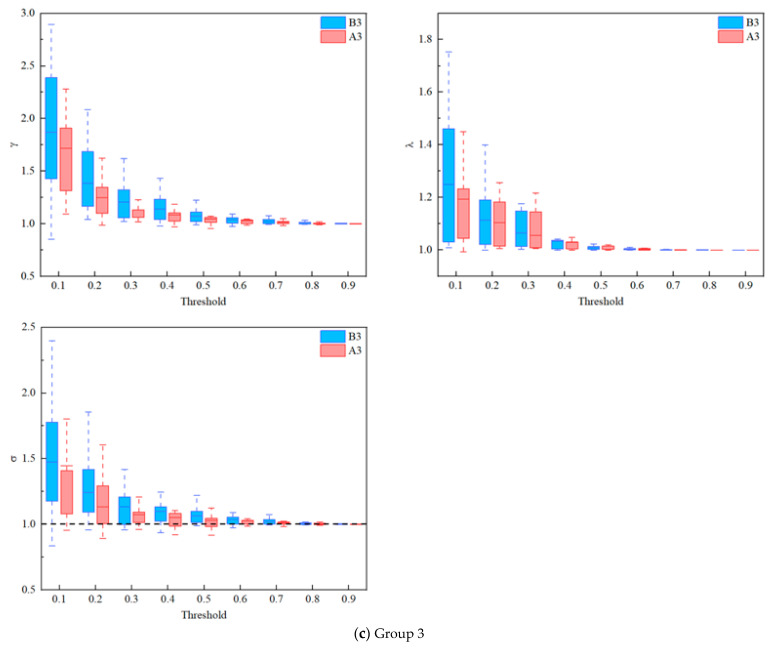
Comparison of the brain network among 3 shifts coal mine workers between before and after shift work: small worldness (γ, λ, σ). (**a**) Group 1. (**b**) Group 2. (**c**) Group 3.

The small-world analysis results for three groups are displayed in Figure 6. Similar to the results of previous studies, the small-world parameters (γ, λ, σ) decreased with increasing thresholds for all three groups of coal mine workers before and after their work shifts [51,59,60]. In addition, all the σ≫1 represented a small-world property of the resting-state brain networks of all three groups of coal mine workers before and after their shifts.

The oxy-Hb-based group differences in betweenness centrality and nodal local efficiency during the resting state were available in Table 4 and Table 5, provided with a paired *t*-test (*p* < 0.05). The results showed that there were significant differences between before and after work shifts in group 1 and group 3, while there were no differences before and after work shifts in group 2 (*p* < 0.05). In particular, CH15 (belong to FPC) passed the paired *t*-test in betweenness centrality in group 1 (*p* = 0.0483) and CH19 (belong to OFC) in group 3 (*p* = 0.039). Further, CH18 (belong to dlPFC) passed the paired *t*-test in nodal local efficiency in group 1 (*p* = 0.0395) and CH14 (belong to dlPFC) in group 3 (*p* = 0.0475).

## 4. Discussion

In this study, an rs-fNIRS measurement was adopted to test the differences in functional connectivity and brain networks in PFC between before and after the work shifts in the morning shift workers, afternoon shift workers, and night shift workers. To the best of our knowledge, this study is the first to investigate the effect of shift work on coal mine workers’ mental states from a brain science perspective. Firstly, the coal mine workers’ brain functional connectivity was significantly correlated with marital status (*p* = 0.038) and education information (*p* = 0.019). Secondly, there were significant differences in the before and after work shift mental states of coal mine workers among three shifts (*p* < 0.05). In particular, for the morning and afternoon shifts coal mine workers, after work shifts’ functional connectivity was decreased than before the work shift (*p* < 0.05). The results showed a significant difference in *COR* matrices between before and after the work shift of the morning shift coal mine workers in the brain region of dlPFC, dlPFC-FPC, dlPFC-OFC, and OFC. For the afternoon shift coal mine workers, the differences in *COR* matrices before and after work shifts were mainly concentrated in the brain region of dlPFC, dlPFC-FPC, FPC, FPC-OFC, and dlPFC-OFC. Further, compared to the morning and afternoon shift workers, the functional connectivity results for the night shift coal mine workers were opposite (*p* < 0.05). The *COR* matrices were significantly increased in the brain region of dlPFC-FPC, FPC, and dlPFC after the work shifts than that before the work shifts. Thirdly, we also discovered that all the states of the morning, afternoon, and night coal mine shift workers exhibited small-world properties in the PFC. Fourthly, significant differences were found in betweenness centrality and nodal local efficiency between before and after the work shifts, both in the morning and night shift workers (*p* < 0.05).

Among the demographic characteristics, marital status and education information may influence the coal mine workers’ brain functional connectivity. Functional connectivity intensity in PFC is significantly associated with cognitive function [61]. Similar to previous studies, low educational attainment or a correlate predicts cognitive decline [25,61]. Compared with married people, it might be easier to suffer from diseases related to a cognitive impairment, such as Alzheimer’s disease, for single and divorced people [62].

The results of our experiments demonstrate that the cognitive function was significantly different before the work shift to the end of the shift in these three shifts of coal mine workers. Therein, the cognitive function performance was lowest among the night shift coal mine workers, followed by the afternoon shift and finally the morning shift, which is consistent with previous studies [14,15]. It has already been demonstrated that the night shift workers experience more fatigue than the morning and afternoon shift workers [63]. Similar to nurses, at the end of the night shift, physical and mental fatigue and cognitive function of coal mine workers were severely impaired, which may lead to operator errors and unsafe behavior [16,64,65].

Concerning the connectivity patterns of B1 and A1, our analysis demonstrated that the resting-state functional connectivity of B1 in the dlPFC, dlPFC-FPC, dlPFC-OFC, and OFC were connected more intensively. It is well accepted that PFC is the key region of complex cognitive control. Specifically, dlPFC mediates executive functions such as planning, working memory, monitoring, selective attention, and inhibiting pre-programmed behavior [66,67,68]. The FPC is crucial for complex human cognitive abilities such as multitasking ability [69]. The OFC is involved in controlling and correcting reward-related and punishment-related behavior, and thus in emotion and touch [70]. This means that after the work shifts, group 1 was more easily experienced a decline in concentration, accompanied by reduced multitasking skills and emotional control.

Similar to group 1, the resting-state functional connectivity of B2 was stronger than that of A2 in the dlPFC, dlPFC-FPC, FPC, FPC-OFC, and dlPFC-OFC. In other words, after the work shifts, the executive functions, complex cognitive abilities, and emotion and touch perception were declined in group 2. Consistent with the results of behavioral experiments and psychometric scales by Reza Kazemi et al., our study showed a decreased cognitive performance of coal mine workers at the end of the morning shifts [13]. On the same lines, behavioral experiments by Azam Esmaily et al. showed that shift work can affect the cognitive function (working memory and attention) of nurses [15]. Lower cognitive performance such as poor concentration, fatigue, and sleepiness can trigger the near-miss events that lead to the unsafe behavior of coal mine workers [65].

It was found that the functional connectivity of group 3 was increased from before and to the end of the shift. The main differences were concentrated in the dlPFC-FPC, FPC, and dlPFC. That is, improved executive functions, including attention, working memory, and multitasking, were found after the work shift in group 3 compared to before their shift. In line with the EEG study, participants with distracted attention had stronger functional brain connectivity intensity [71]. Interestingly, an experiment by Azam Esmaily Reza Kazemi et al. demonstrated that among the three shifts, the cognitive function was decreased most prominently after the night shift [15]. Our results are different from the previous report, perhaps because the work content of the night shift is different from the morning and afternoon shifts. According to the regulations of Hongliulin Coal Company, the morning and afternoon shifts are coal mining production shifts, while the night shift is the maintenance and repair shifts. Compared to the production shift, the working environment of the maintenance and repair shift is relatively good, without production noise and dust. The effects of different work contents and work environments on the cognitive function of three-shift coal mine workers can be further investigated in the future.

Based on these analyses, the brain network differences among the three groups were analyzed. The results showed that the trends in $C_{net}$, $E_{global}$, $E_{loc},$ and $L_{p}$ of all the states among the 3 groups under 10 thresholds were in line with previous studies, with no significant differences between before and after shifts [50,59,72]. Further, the small-world properties of the brain networks of all the states among the three groups were calculated separately, and the results were greater than 1. That is, the rs-fNIRS brain network data of all groups exhibited small-world properties.

Notably, the results of the two-sample *t*-test showed that there were significant differences between in betweenness centrality and nodal local efficiency before and after work shifts in group 1 and group 3, while there were no differences before and after work shifts in group 2. Concretely, the significant differences for betweenness centrality in the FPC in group 1. Cortically, the larger betweenness centrality indicates the greater influence of the target region as a hub within the brain network [73]. That is, the multitasking ability was significantly different between B1 and A1. It coincides with the study by Marja-Leena Haavisto Reza Kazemi et al. that shift work can impair multitasking performance [74]. In addition, there were also significant differences for betweenness centrality in the OFC in group 3, which may indicate that emotion control was reduced in the night shift work. Our results were in line with the study by Dov Zohar Reza Kazemi et al. that sleep loss impaired emotional reactivity [75]. Moreover, the significant differences in nodal local efficiency in the dlPFC in both group 1 and group 3. The higher nodal local efficiency represents better efficiency of information transformation within a local subgraph consisting of only the neighbors of a given node [57]. Consistent with the results of the study by Ann Rhéaume Reza Kazemi et al., cognitive performance was declined after long work hours and shift work [65].

As far as we know, our study is a preliminary one, drawing attention to the correlation between cognitive functions and shift work in coal mine workers in the real field from the perspective of brain science. In addition, this research has some limitations. First, the sample size of this study was relatively small. Second, the tests after shifts may have been affected by a time constraint. Due to the special working conditions of coal mine workers underground, the experiments were conducted after the miners had gone up the shaft and washed up. Third, the present study only observed the brain functional connectivity of the same miners before and after the work shift. In the future, the same subjects could be observed continuously for multiple days to further explore the effect of shift work on the cognitive abilities of coal mine workers.

## 5. Conclusions

The results of this study show that working a shift for an extended period can significantly affect cognitive function among coal mine workers. On the one hand, the results showed that there were significant differences in cognitive ability between before and after the work shifts among the three-shift workers. The brain functional connectivity was reduced ranking during the night, afternoon, and morning shifts. Decreased brain functional connectivity at the end of the working shift was found compared with before in the morning and afternoon shifts. Opposite results were obtained during the night shift. On the other hand, significant differences were found in betweenness centrality and nodal local efficiency in the morning and the night shift. The current findings also provide new insights regarding the effect of shift work on the cognitive ability of Chinses coal mine workers. Ideally, combining fNIRS with psychological scales and behavior experiments can be used to investigate the influence of shift work on cognitive function among coal mine workers in future studies.

## Figures and Tables

**Table 1:** The demographic information of 54 coal mine workers.

	Mean ± Std				Chi-Square Test		One-Way ANOVA	
	Total (*n* = 54)	Group 1 (*n* = 17)	Group 2 (*n* = 18)	Group 3 (*n* = 19)	$\chi^{2}$	$p_{1}$	$p_{2}$	*F*
Length of service/year	9.91 ± 7.81	10.29 ± 6.72	11.9 ± 8.39	7.68 ± 7.33	577.785	0.92	0.704	0.788
Height/cm	172.63 ± 4.71	174 ± 4.78	171.78 ± 3.41	171.42 ± 4.86	306.464	0.34	0.316	1.215
Age/year	36.06 ± 7.42	36.8 ± 6.77	37.5 ± 7.61	34 ± 7.13	102.72	0.903	0.542	0.724
Weight/kg	69.93 ± 8.36	69.4 ± 7.99	71.9 ± 5.36	68.5 ± 10.2	99.392	0.924	0.132	1.959
Marital status	-	-	-	-	38.096	0.086	0.038 *	4.519
Education information	-	-	-	-	170.959	0.417	0.019 *	3.246

Note: group 1 represents morning shift workers, group 2 represents afternoon shift workers, and group 3 represents night shift workers; * represents passed the 95% One-way ANOVA test.

**Table 2:** The marital status and education information of 54 coal mine workers.

	Total (*n* = 54)		Group 1 (*n* = 17)		Group 2 (*n* = 18)		Group 3 (*n* = 19)	
	*n*	%	*n*	%	*n*	%	*n*	%
Marital status								
Married	48	88.9	16	94.1	17	94.4	15	78.9
Unmarried	6	11.1	1	5.9	1	5.6	4	21.1
Education information								
Bachelor’s degree	4	7.4	1	5.9	2	11.1	1	5.3
College	12	22.2	5	29.4	3	16.7	4	21.1
High school	27	50.0	5	29.4	11	61.1	11	57.9
Junior high school	1	1.9	1	5.9	0	0.0	0	0.0
Technical secondary school	10	18.5	5	29.4	2	11.1	3	15.8

Note: group 1 represents morning shift workers, group 2 represents afternoon shift workers, and group 3 represents night shift workers.

**Table 3:** Mean locations of 22 fNIRS channels.

CH	Brodmann Area	MNI Coordinates			
		x	y	z	
CH01	9—Dorsolateral prefrontal cortex	34	45	43	0.85714
CH02	9—Dorsolateral prefrontal cortex	14	55	44	1
CH03	9—Dorsolateral prefrontal cortex	−9	55	45	1
CH04	9—Dorsolateral prefrontal cortex	−28	45	43	0.90498
CH05	46—Dorsolateral prefrontal cortex	43	47	30	0.60444
CH06	46—Dorsolateral prefrontal cortex	25	61	31	0.35887
CH07	10—Frontopolar cortex	4	63	32	0.75357
CH08	9—Dorsolateral prefrontal cortex	−19	60	33	0.44939
CH09	46—Dorsolateral prefrontal cortex	−40	47	30	0.67544
CH10	10—Frontopolar cortex	35	62	20	0.51373
CH11	10—Frontopolar cortex	15	70	21	1
CH12	10—Frontopolar cortex	−10	70	20	1
CH13	10—Frontopolar cortex	−32	62	19	0.51538
CH14	46—Dorsolateral prefrontal cortex	45	58	6	0.59144
CH15	10—Frontopolar cortex	25	71	8	0.83502
CH16	10—Frontopolar cortex	5	72	9	1
CH17	10—Frontopolar cortex	−21	71	8	0.88372
CH18	46—Dorsolateral prefrontal cortex	−42	58	6	0.60311
CH19	11—Orbitofrontal cortex	34	67	−6	0.59649
CH20	11—Orbitofrontal cortex	15	73	−4	0.52229
CH21	11—Orbitofrontal cortex	−11	73	−3	0.47
CH22	11—Orbitofrontal cortex	−32	66	−4	0.51408

**Table 4:** Group differences in betweenness centrality during resting state.

CH	Brodmann Area	Betweenness Centrality							
		Mean ± Std		*p*_1_	*T*_1_	Mean ± Std		*p*_2_	*T*_2_
		B1	A1			B3	A3		
CH01	9—Dorsolateral prefrontal cortex	3.97 ± 0.37	4.05 ± 0.41	0.9565	−0.05	2.37 ± 0.02	3.77 ± 0.03	0.1228	−1.5803
CH02	9—Dorsolateral prefrontal cortex	3.58 ± 0.33	2.73 ± 0.29	0.446	0.77	3.3 ± 0.03	2.15 ± 0.01	0.1478	1.479
CH03	9—Dorsolateral prefrontal cortex	1.17 ± 0.16	1.72 ± 0.23	0.436	−0.79	3.1 ± 0.04	1.45 ± 0.01	0.0807	1.7969
CH04	9—Dorsolateral prefrontal cortex	5.16 ± 0.36	6.51 ± 0.63	0.4633	−0.74	4.47 ± 0.03	3.69 ± 0.02	0.4066	0.8398
CH05	46—Dorsolateral prefrontal cortex	3.29 ± 0.37	2.44 ± 0.3	0.485	0.71	1.75 ± 0.02	1.54 ± 0.02	0.7618	0.3054
CH06	46—Dorsolateral prefrontal cortex	6.71 ± 0.42	5.86 ± 0.5	0.6064	0.52	5.32 ± 0.04	6.06 ± 0.06	0.6684	−0.4318
CH07	10—Frontopolar area	2.18 ± 0.25	2.67 ± 0.26	0.592	−0.54	1.22 ± 0.01	0.74 ± 0.01	0.1802	1.3667
CH08	9—Dorsolateral prefrontal cortex	5.04 ± 0.33	5.24 ± 0.48	0.8914	−0.14	4.27 ± 0.03	2.91 ± 0.01	0.0911	1.7361
CH09	46—Dorsolateral prefrontal cortex	2.42 ± 0.45	2.51 ± 0.23	0.944	−0.07	1.49 ± 0.02	1.38 ± 0.02	0.8736	0.1602
CH10	10—Frontopolar area	5.42 ± 0.37	4.23 ± 0.3	0.3218	1.01	5.26 ± 0.03	4.76 ± 0.03	0.6606	0.4427
CH11	10—Frontopolar area	4.57 ± 0.27	4.61 ± 0.35	0.9747	−0.03	4.44 ± 0.04	4.63 ± 0.03	0.8701	−0.1647
CH12	10—Frontopolar area	3.65 ± 0.32	4.76 ± 0.23	0.2711	−1.12	4.83 ± 0.04	4.48 ± 0.03	0.7832	0.2772
CH13	10—Frontopolar area	6.39 ± 0.48	6.21 ± 0.42	0.9082	0.12	5.74 ± 0.03	6.82 ± 0.04	0.3976	−0.8562
CH14	46—Dorsolateral prefrontal cortex	3.23 ± 0.38	1.53 ± 0.15	0.1063	1.66	3.28 ± 0.02	2.77 ± 0.04	0.6566	0.4483
CH15	10—Frontopolar area	1.46 ± 0.15	3.04 ± 0.27	0.0483 *	−2.05	1.41 ± 0.02	1.98 ± 0.03	0.5464	−0.609
CH16	10—Frontopolar area	6.11 ± 0.64	4.63 ± 0.44	0.4503	0.76	5 ± 0.03	4.89 ± 0.03	0.9157	0.1066
CH17	10—Frontopolar area	1.05 ± 0.16	2.26 ± 0.29	0.1529	−1.46	0.64 ± 0.01	1.2 ± 0.01	0.1257	−1.5676
CH18	46—Dorsolateral prefrontal cortex	4.66 ± 0.35	4.17 ± 0.33	0.6849	0.41	4.55 ± 0.03	4.72 ± 0.03	0.8773	−0.1554
CH19	11—Orbitofrontal area	2.37 ± 0.22	4.47 ± 0.49	0.1256	−1.57	3.88 ± 0.04	1.54 ± 0.02	0.039 *	2.1422
CH20	11—Orbitofrontal area	5.74 ± 0.48	4.89 ± 0.44	0.6048	0.52	4.51 ± 0.02	5.16 ± 0.03	0.4825	−0.7097
CH21	11—Orbitofrontal area	1.97 ± 0.39	1.51 ± 0.16	0.6635	0.44	1.68 ± 0.03	1.52 ± 0.03	0.8672	0.1684
CH22	11—Orbitofrontal area	1.2 ± 0.1	2.98 ± 0.54	0.2058	−1.29	1.33 ± 0.02	0.61 ± 0.01	0.1884	1.3408

* represents passed the 95% paired *t*-test.

**Table 5:** Group differences in nodal local efficiency during resting state.

	Brodmann Area	Nodal Local Efficiency							
		Mean ± Std		*p*_1_	*T*_1_	Mean ± Std		*p*_2_	*T*_2_
		B1	A1			B3	A3		
CH01	9—Dorsolateral prefrontal cortex	0.61 ± 0.1	0.6 ± 0.16	0.8661	0.1699	0.68 ± 0.07	0.68 ± 0.09	0.9215	−0.0992
CH02	9—Dorsolateral prefrontal cortex	0.64 ± 0.14	0.66 ± 0.15	0.7979	−0.2582	0.71 ± 0.04	0.69 ± 0.08	0.5201	0.6496
CH03	9—Dorsolateral prefrontal cortex	0.64 ± 0.16	0.66 ± 0.14	0.7127	−0.3715	0.66 ± 0.12	0.68 ± 0.08	0.5706	−0.5724
CH04	9—Dorsolateral prefrontal cortex	0.68 ± 0.06	0.62 ± 0.12	0.0673	1.8938	0.68 ± 0.03	0.69 ± 0.07	0.892	−0.1367
CH05	46—Dorsolateral prefrontal cortex	0.6 ± 0.14	0.62 ± 0.16	0.6814	−0.4143	0.52 ± 0.2	0.46 ± 0.23	0.4284	0.8011
CH06	46—Dorsolateral prefrontal cortex	0.67 ± 0.05	0.67 ± 0.09	0.8543	−0.1851	0.69 ± 0.04	0.7 ± 0.06	0.7372	−0.3382
CH07	10—Frontopolar area	0.62 ± 0.15	0.65 ± 0.13	0.5586	−0.5911	0.65 ± 0.15	0.67 ± 0.1	0.4791	−0.7153
CH08	9—Dorsolateral prefrontal cortex	0.7 ± 0.05	0.64 ± 0.1	0.0444	2.0926	0.69 ± 0.07	0.69 ± 0.08	0.9294	0.0892
CH09	46—Dorsolateral prefrontal cortex	0.58 ± 0.17	0.6 ± 0.11	0.5835	−0.5539	0.51 ± 0.2	0.46 ± 0.27	0.5196	0.6504
CH10	10—Frontopolar area	0.68 ± 0.05	0.68 ± 0.05	0.8924	0.1363	0.69 ± 0.05	0.67 ± 0.06	0.2535	1.1605
CH11	10—Frontopolar area	0.69 ± 0.05	0.68 ± 0.07	0.5912	0.5426	0.7 ± 0.07	0.7 ± 0.04	0.8454	−0.1963
CH12	10—Frontopolar area	0.71 ± 0.06	0.7 ± 0.03	0.5628	0.5848	0.69 ± 0.07	0.71 ± 0.04	0.2126	−1.2688
CH13	10—Frontopolar area	0.65 ± 0.06	0.66 ± 0.06	0.803	−0.2515	0.69 ± 0.05	0.68 ± 0.04	0.5956	0.5356
CH14	46—Dorsolateral prefrontal cortex	0.56 ± 0.14	0.59 ± 0.17	0.5213	−0.6484	0.63 ± 0.09	0.55 ± 0.14	0.0475 *	2.0521
CH15	10—Frontopolar area	0.55 ± 0.15	0.53 ± 0.17	0.7054	0.3814	0.43 ± 0.21	0.44 ± 0.21	0.8407	−0.2025
CH16	10—Frontopolar area	0.67 ± 0.07	0.68 ± 0.06	0.5587	−0.591	0.67 ± 0.07	0.66 ± 0.1	0.6849	0.4091
CH17	10—Frontopolar area	0.39 ± 0.23	0.46 ± 0.22	0.3337	−0.9815	0.37 ± 0.22	0.43 ± 0.18	0.3565	−0.9341
CH18	46—Dorsolateral prefrontal cortex	0.54 ± 0.14	0.63 ± 0.08	0.0395 *	−2.1468	0.6 ± 0.11	0.59 ± 0.11	0.8275	0.2195
CH19	11—Orbitofrontal area	0.56 ± 0.13	0.5 ± 0.17	0.2697	1.1232	0.55 ± 0.12	0.45 ± 0.19	0.0701	1.8666
CH20	11—Orbitofrontal area	0.65 ± 0.07	0.64 ± 0.14	0.8883	0.1416	0.62 ± 0.08	0.62 ± 0.1	0.9437	0.0711
CH21	11—Orbitofrontal area	0.5 ± 0.22	0.49 ± 0.23	0.858	0.1804	0.5 ± 0.17	0.58 ± 0.13	0.1736	−1.3882
CH22	11—Orbitofrontal area	0.59 ± 0.18	0.48 ± 0.19	0.1006	1.6908	0.45 ± 0.18	0.41 ± 0.19	0.5917	0.5411

* represents passed the 95% paired *t*-test.

## Data Availability

The data that support the findings of this study are available from the corresponding author upon reasonable request.
